# Incidence of tuberous sclerosis and age at first diagnosis: new data and emerging trends from a national, prospective surveillance study

**DOI:** 10.1186/s13023-018-0870-y

**Published:** 2018-07-17

**Authors:** Daniel Ebrahimi-Fakhari, Lilian Lisa Mann, Martin Poryo, Norbert Graf, Rüdiger von Kries, Beate Heinrich, Darius Ebrahimi-Fakhari, Marina Flotats-Bastardas, Ludwig Gortner, Michael Zemlin, Sascha Meyer

**Affiliations:** 1 Department of Pediatric Neurology, Saarland University Medical Center, Building 9, Kirrberger Strasse, 66421 Homburg, Saarland Germany; 2 Department of Pediatric Cardiology, Saarland University Medical Center, Homburg, Germany; 3 Department of Pediatric Oncology and Hematology, Saarland University Medical Center, Homburg, Germany; 4 Division of Epidemiology, Institute of Social Pediatrics and Adolescent Medicine, Ludwig Maximilian’s University, Munich, Germany; 5 German Paediatric Surveillance Unit (ESPED), Coordination Center for Clinical Studies, Heinrich Heine University, Düsseldorf, Germany; 6 Department of Neurology, Boston Children’s Hospital, Harvard Medical School, Boston, MA USA

**Keywords:** Epidemiology, Incidence, Tuberous sclerosis, Everolimus, mTOR, Prenatal rhabdomyoma, hamartomas, Neurologic manifestations, Infantile spasms, Seizures

## Abstract

**Background:**

Tuberous Sclerosis Complex (TSC) is a rare multisystem disorder. In 2012 diagnostic criteria for TSC were revised. However, data on the incidence of TSC are limited.

**Methods:**

Prospective, national surveillance study in Germany over a 2-year-period (03/2015–02/2017) using current revised criteria for TSC. Patients up to the age of 18 years with a new diagnosis of definite or possible TSC (clinical and/or genetic) were included. The aims of this study were 1) to generate up-to-date data on the incidence of definite or possible TSC, 2) to assess age at first diagnosis, and 3) to compare these data with previous epidemiologic data.

**Results:**

In total, 86 patients met inclusion criteria (definite or possible TSC) with a median age at diagnosis of 6 months (range: 5 months before birth – 197 months of age). Among patients identified with features of TSC, 73.3% met criteria for definite diagnosis (median age: 7 months) and 26.7% met criteria for a possible diagnosis (median age: 3 months). 55.8% of patients were male. When excluding prenatally diagnosed patients, median age at diagnosis was 11 months with a range of 0 to 197 months. The 3 most common clinical features at diagnosis of TSC were central nervous system involvement in 73.3% patients (of these 95.2% experienced seizures), cutaneous involvement in 58.1% patients (with the most common lesion being hypomelanotic macules in 92%) and cardiac rhabdomyoma in half of the patients. Cardiac rhabdomyoma were detected by prenatal ultrasonography in 22.1% of patients. The presence of cardiac rhabdomyoma was associated with cardiac arrhythmias in 25.6% (about 13% of all diagnosed patients) in our cohort. The overall prevalence of seizure disorders was 69.8%. The annual incidence rate of TSC is estimated at a minimum of 1:17.785 live births. However correcting for underreporting, the estimated incidence rate of definite or possible TSC is approximately 1:6.760–1:13.520 live births in Germany.

**Conclusions:**

This is the first study that assessed prospectively the incidence rate of TSC in children and adolescents using the updated diagnostic criteria of 2012. This prospective surveillance study demonstrates a low age at first diagnosis (median: 6 months), likely due to antenatal detection of cardiac rhabdomyoma. Early diagnosis bears the potential for implementing effective therapies at an earlier stage.

**Electronic supplementary material:**

The online version of this article (10.1186/s13023-018-0870-y) contains supplementary material, which is available to authorized users.

## Background

Tuberous Sclerosis Complex (TSC) is a rare genetic neurocutaneous, multisystem disorder with a variable clinical phenotype [1–3]. It is characterized by autosomal-dominant mutations in the *TSC1* or *TSC2* genes (encoding for the protein Hamartin on chromosome 9q34 and Tuberin on chromosome 16q13 respectively) [4–6], leading to overactivation of the mTOR (mechanistic target of rapamycin) pathway with increased cell proliferation and a range of other consequences [7]. Benign tumor growth represents the hallmark of the disease with the central nervous system (CNS), the kidney and the skin being the most commonly affected organs. TSC features develop in an age dependent manner [8]. In 2012, the Tuberous Sclerosis Consensus Conference updated diagnostic criteria and surveillance management of the disease (Table 1) [9, 10].

**Table 1:** Diagnostic criteria according to the 2012 International Tuberous Sclerosis Complex Consensus Conference [9]

Definite diagnosis: Two major diagnostic criteria or one major with greater than or equal two minor diagnostic criteria or the presence of a *TSC1* or *TSC2* mutation (of confirmed pathogenicity^a^) Possible diagnosis: Either one major diagnostic criteria or greater than or equal two minor diagnostic criteria	
Major criteria:	• Cortical dysplasias (incl. tubers and cerebral white matter radial migration lines) • Subependymal nodules (SEN) • Subependymal giant cell astrocytoma (SEGA) • Cardiac rhabdomyoma • Hypomelanotic macules (≥3, at least 5 mm diameter) • Angiofibromas (*n* ≥ 3) or fibrous cephalic plaque • Ungual fibromas (≥2) • Shagreen patch • Angiomyolipomas (≥2) ^b, c^ • Lymphangioleiomyomatosis (LAM) ^b^ • Multiple retinal hamartomas
Minor criteria:	• ´Confetti´ skin lesions • Dental enamel pits (> 3) • Intraoral fibromas (≥2) • Multiple renal cysts • Retinal achromatic patch • Nonrenal hamartomas
Genetics:	Identification of either a *TSC1* or *TSC2* pathogenic mutation in DNA from normal tissue^a^

(From: Northrup H, Krueger DA, on behalf of the International Tuberous Sclerosis Complex Consensus Group. Tuberous Sclerosis Complex Diagnostic Criteria Update: Recommendations of the 2012 International Tuberous Sclerosis Complex Consensus Conference. Pediatr Neurol 2013; 49: 243–254. © The authors. License Number 4341381420907)

^a^ Pathogenic mutation: a mutation that clearly inactivates the function of the TSC1 or TSC2 proteins (e.g., out-of-frame indel or nonsense mutation), prevents protein synthesis (e.g., large genomic deletion), or is a missense mutation whose effect on protein function has been established by functional assessment (www.lovd.nl/TSC1, www.lovd/nl/TSC2 and Hoogeveen-Westerveld et al., 2012 and 2013). Other TSC1 or TSC2 variants whose effect on function is less certain do not meet these criteria, and are not sufficient to make a definite diagnosis of TSC. Note that 10 to 25% of TSC patients have no mutation identified by conventional genetic testing, and a normal result does not exclude TSC, or have any effect on the use of clinical diagnostic criteria to diagnose TSC

^b^ A combination of the two major clinical features (lymphangioleiomyomatosis and angiomyolipomas) without other features does not meet criteria for a definite diagnosis

^c^ Angiomyolipomas might also occur in the liver or other organ systems

In recent years large-scale data on the clinical and genetic characteristics have emerged, most importantly from the TOSCA (TuberOus SClerosis registry to increase disease Awareness) study. The TOSCA study is a large natural history study encompassing 2093 patients with TSC [11]. In this registry, the median age of diagnosis of TSC was one year (range 0–69). In 5.9% patients, the diagnosis was made antenatally. While cardiac rhabdomyoma were found in 34.3% patients, mean age of diagnosis of cardiac rhabdomyoma was 3.1 years. Whilst knowledge of clinical and genetic features of TSC has increased and treatment modalities have been established, there is still a lack of prospective studies on the incidence of TSC [11–16].

The German Paediatric Surveillance Unit (ESPED) was founded in 1992 to generate incidence data and detailed clinical descriptions of rare, childhood-onset diseases in Germany requiring in-hospital treatment [17, 18]. Electronic mailing cards are sent monthly to the heads of all pediatric departments in Germany asking whether a patient was newly diagnosed with one of the 12 rare clinical conditions currently under review (active surveillance system, i.e. negative reporting (no cases) as well) [19]. In case of a positive answer, a detailed questionnaire is sent to the reporting hospital by ESPED. Survey periods usually last two years. In addition to epidemiological data, further information from extended laboratory testing or genetic analyses can be integrated into individual research projects. Between 1992 and 2017, ESPED completed 96 prospective studies on rare diseases in children [18]. Thus, ESPED surveys are an important contributor in the field of clinical epidemiology in children with rare diseases [19].

The major aims of this prospective, national surveillance study were:

1. To generate up-to-date data on the incidence of definite or possible TSC in Germany over a 2-year-period using current revised criteria for TSC
2. To assess age at first diagnosis, and
3. To compare our results with previous epidemiologic data

## Methods

This study was a prospective, national surveillance study conducted from March 1, 2015 to February 28, 2017 in Germany. The study was approved by the Institutional Ethics Review Board of Saarland, Germany (file no. 219/14). Patients up to the age of 18 years with a new diagnosis of definite or possible TSC [9] (clinical and/or genetic) were prospectively included.

Electronic and postal questionnaires (see Additional file 1) were sent monthly to all departments of pediatrics (*n* = 349), all social pediatric centers (*n* = 120) and TSC centers (*n* = 18) in Germany, using the German Paediatric Surveillance Unit for Rare Diseases (ESPED) system [17–19]. Statistical analysis was performed using IBM SPSS Statistics version 24 (IBM, Armonk, NY, USA). Descriptive data are presented as median and range. The incidence rate of TSC is estimated from the number of live births in Germany (737.575 in 2015 and 792.000 in 2016) during the study period. For estimating the potential size of underreporting, we used estimates from previous ESPED studies with correction factors for completeness between 0.38–0.76 [17, 18].

For classification of patients with a definite or possible diagnosis of TSC, we used the current Tuberous Sclerosis Consensus Conference updated diagnostic guidelines [9].

## Results

**Fig. 1:**
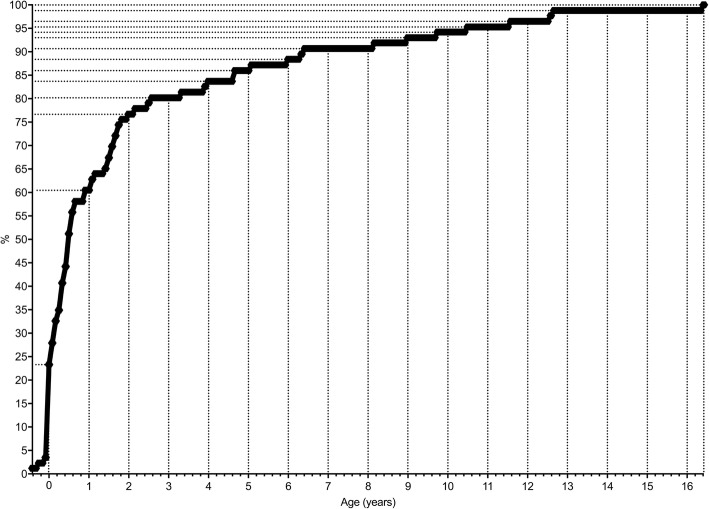
Cumulative age distribution at first diagnosis

Initially 150 cases were reported to ESPED, from which 135 patient questionnaires were received (response rate 90%). 40 questionnaires did not report TSC patients and 9 contained redundant datasets (double reporting) and were excluded. In total, 86 patients met inclusion criteria. Median age at diagnosis (definite or possible TSC) was 6 months (range: 5 months before birth – 197 months of age). Among patients identified with features of TSC, 73.3% (63/86) met criteria for definite diagnosis (median age: 7 months; range: 5 months before birth – 139 months of age) and 26.7% (23/86) met criteria for a possible diagnosis (median age: 3 months; range: 1 month before birth – 197 months of age). 55.8% (48/86) of patients were male. Age at diagnosis is shown in Fig. 1. When excluding prenatally diagnosed patients (19/86), median age at diagnosis (definite or possible TSC) was 11 months with a range of 0 to 197 months.

### Incidence

Based on our findings, the annual incidence rate of TSC (definite or possible TSC) is estimated at a minimum of 1:17.785 live births. However correcting for underreporting using data from previous ESPED analyses, the estimated incidence rate of definite or possible TSC is approximately 1:6.760–1:13.520 live births in Germany.

### Clinical features

#### Antenatal characteristics

Cardiac rhabdomyoma were detected by antenatal ultrasound in 22.1% of patients (19/86), leading to antenatal diagnosis of ‘possible TSC’. One patient had concomitant cerebral abnormalities (no other characteristics were reported).

#### Postnatal characteristics

The most common clinical feature at diagnosis of TSC was central nervous system (CNS) involvement in 73.3% patients (63/86), of these 95.2% (60/63) experienced seizures. Hence, the overall prevalence of seizure disorders in our cohort was 69.8% (60/86). Cutaneous involvement was seen in 58.1% patients (50/86); with the most common lesion being hypomelanotic macules in 92% (46/50). Cardiac rhabdomyoma manifested in half of the patients (43/86), 25.6% of these (11/43) with cardiac arrhythmia.

**Fig. 2:**
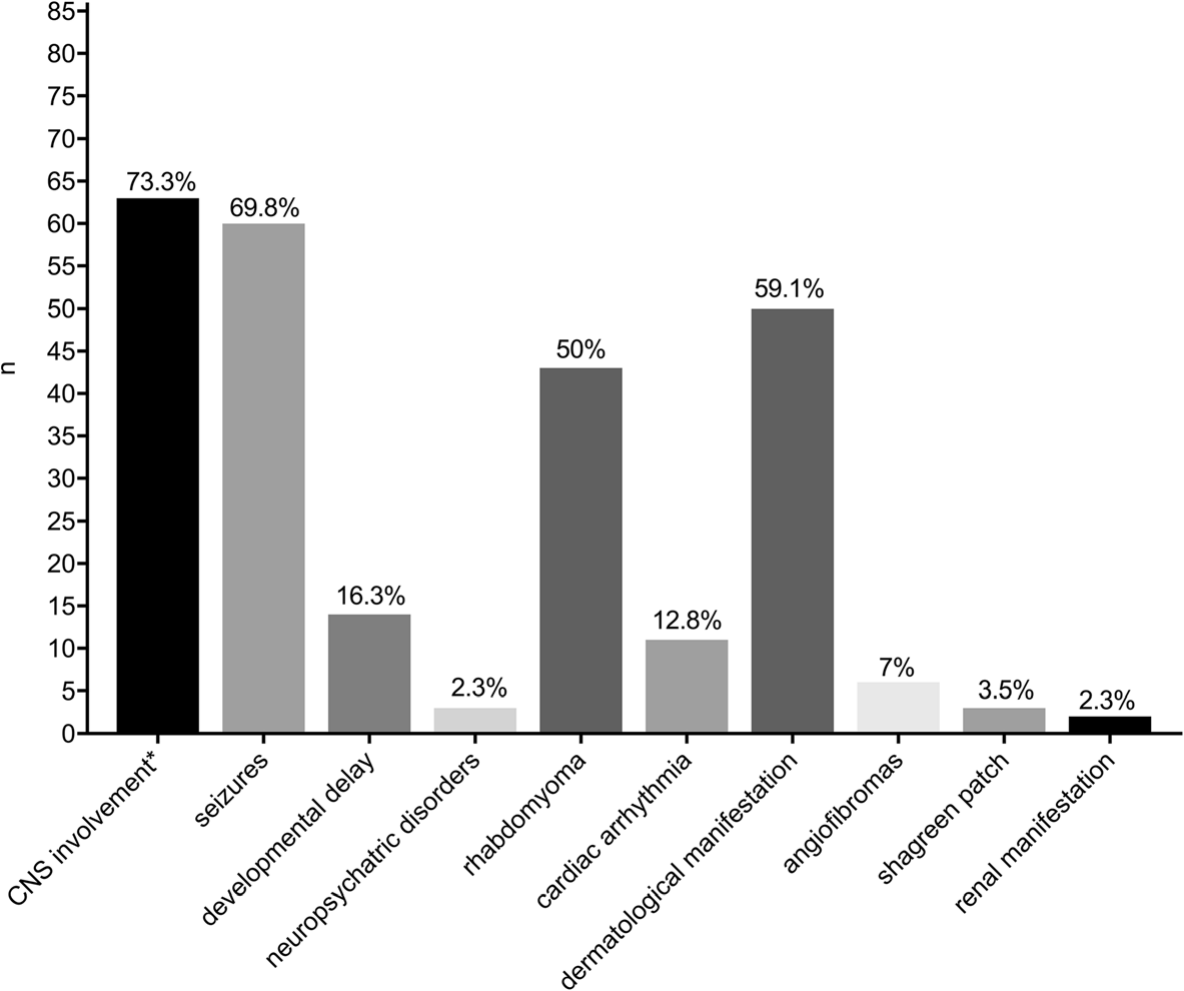
Clinical features leading to first diagnosis. * Including seizures, developmental delay, neuropsychiatric disorders (e.g. autistic characteristics); multiple entries possible. Abbreviations: CNS (central nervous system); (n: number of patients)

**Fig. 3:**
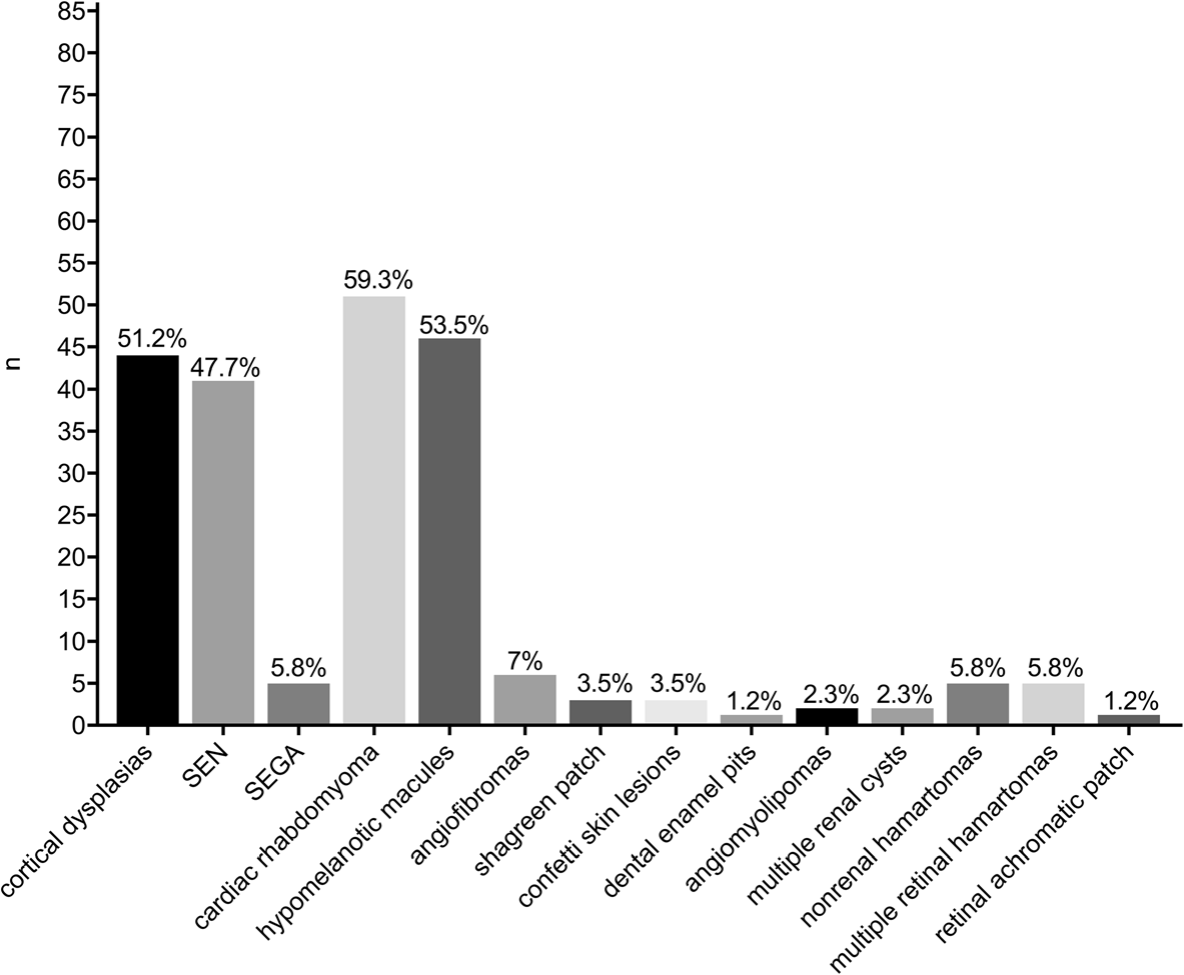
Clinical features after comprehensive diagnostic work-up. The majority of patients presented with CNS involvement (cortical dysplasias 51.5% (44/86); subependymal nodules (SEN) 47.7% (41/86) and subependymal giant cell astrocytoma (SEGA) 5,8% (5/86). Followed by cardiac rhabydomyoma in 59.3% (51/86) and hypomelanotic macules in 53.5% (46/86). The other clinical symptoms were heterogeneous. (n: number of patients)

The spectrum of TSC manifestations other than cardiac, cutaneous and CNS involvement was heterogeneous (Fig. 2). Results from comprehensive diagnostic workup, following surveillance and management recommendations for newly diagnosed or suspected TSC [10] are detailed in Fig. 3.

### Diagnostic tests

**Fig. 4:**
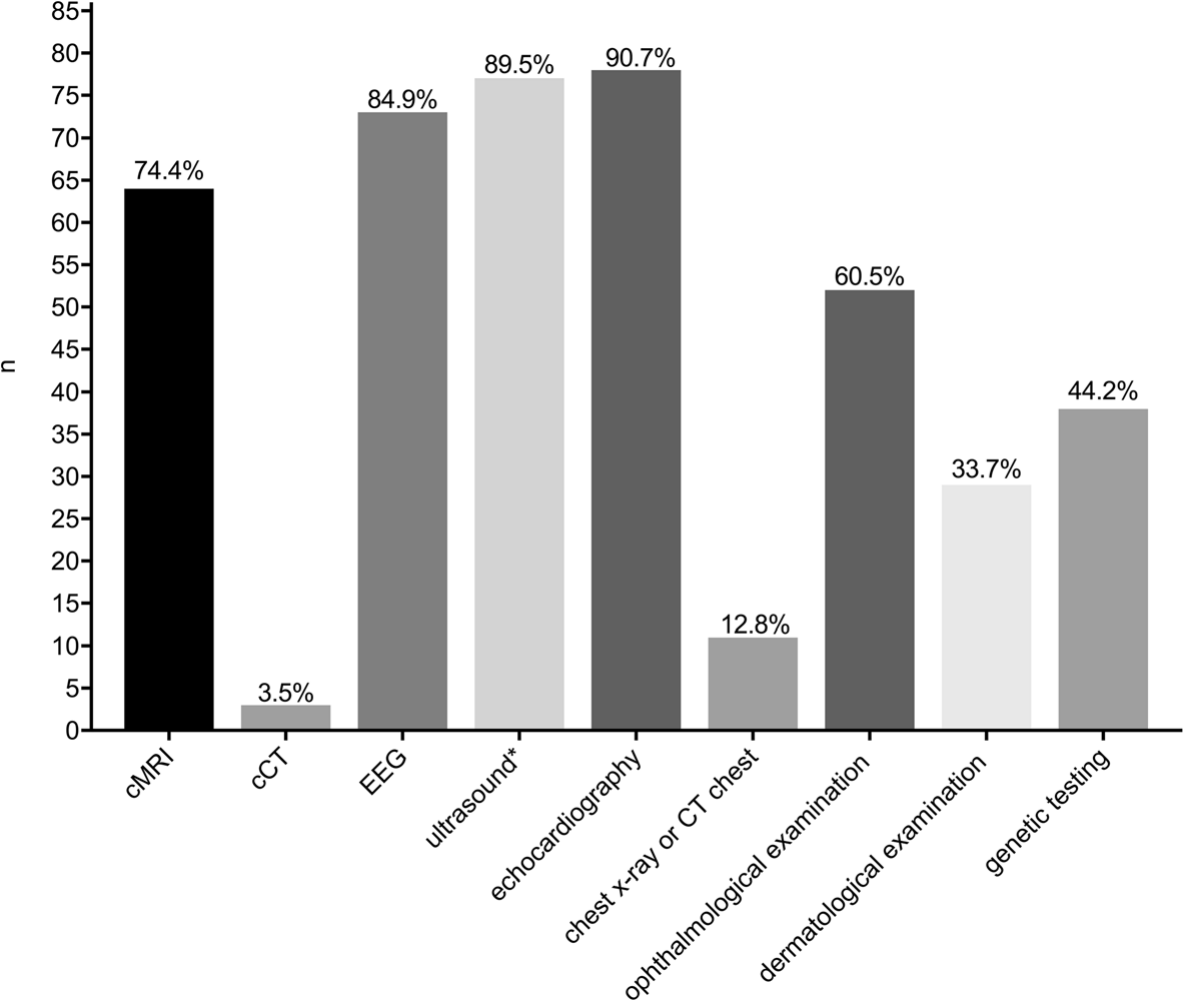
Tests used to establish the diagnosis. The most common diagnostic study performed was echocardiography in 90.7% (78/86), followed by ultrasound (cerebral or abdominal) in 89.5% (77/86). An electroencephalogram (EEG) was performed in 84.9% (73/86). Cranial magnetic resonance imaging (cMRI) was obtained in 74.4% (64/86) as well as cranial CT imaging in 3 patients (3.4%). Formal skin examination was only performed in 33.7% (29/86), while cutaneous involvement was noted in 58.1% of all patients. (n: number of patients)

Tests used to establish the diagnosis are detailed in Fig. 4. The most common diagnostic study performed was echocardiography in 90.7% (78/86), followed by ultrasound (cerebral or abdominal) in 89.5% (77/86). An electroencephalogram (EEG) was performed in 84.9% (73/86) of cases. Cranial magnetic resonance imaging (cMRI) was obtained in 74.4% (64/86) as well as cranial CT imaging in 3 patients (3.4%). A formal skin examination by a dermatologist was only performed in 33.7% (29/86), while cutaneous involvement was noted in 58.1% of all patients.

### Genetics

Results from genetic testing were available in 53.5% patients (46/86): (*TSC1*: 21.7% (10/46); *TSC2:* 58.7% (27/46); no mutation identified (NMI) 19.6% (9/46)). Of note, two patients with *TSC2* mutation also had a *PKD1* mutation (contiguous gene syndrome). A family history of TSC was found in 13 of 86 patients (15.1%).

## Discussion

We here present a prospective epidemiological study that aimed at determining the incidence of TSC, using the current revised diagnostic criteria from 2012 Tuberous Sclerosis Consensus Conference [9]. Although our study provides an estimate of the incidence of definite or possible TSC in Germany based on active surveillance data, we assume that the true incidence is probably still under-estimated for the following reasons:

The true number of TSC patients in our study is unknown. Reporting bias could not be estimated by capture-recapture analysis since no independent second data source was available. We used estimates from previous ESPED studies to estimate a range for potential underreporting (between 0.38–0.76). Interestingly, our incidence data were similar to previous reports, where incidence rates in adults were calculated by employing prevalence data analysis [12]. Our data are also corroborated by a retrospective nationwide cohort study (1997–2010) estimating the incidence of TSC at 0.153 per 100.000 person years in Taiwan [20]. Ascertainment bias remains a possible confounder because of the broad spectrum of disease manifestations and severity, rendering a clinical diagnosis of TSC challenging in mildly affected individuals (e.g. with mosaicism in NMI patients) [21, 22]. In addition, there are TSC patients with NMI (10–15%), in which mosaicism and intronic mutations have only been detected by using next generation sequencing (in 85% of NMI patients) [22]. We acknowledge that these genetic approaches are not yet current standard of care, especially if a definite diagnosis is made using clinical diagnostic criteria. However, with increasing availability of next generation sequencing, early genetic diagnoses will become more common. Genetic testing was not a pre-requisite for study participation; hence results from genetic testing were only available in 53.5% patients. This is comparable to the data from the TOSCA registry, where genetic testing was performed in 43.1% patients [11]. Given the age-related expression of TSC, one shortcoming of our study was that we were not able to assess the number of children with a possible diagnosis who would eventually develop a definite diagnosis of TSC, thus potentially overestimating the true incidence of TSC. When comparing our incidence results with published work on TSC incidence, it is important to note that in the report by *Osborne* et al. from 1993 a different set of diagnostic criteria was used. In this study cases were classified as “definitive” and “presumptive” to generate incidence data [13]. These two different approaches may have contributed to differences in incidence rates between the two studies [13].

TSC is a very heterogeneous disorder both with regard to age-related expression and variability of clinical manifestations [1]. Of note, age at first diagnosis in our study was substantially lower than in previous epidemiological reports [11, 16], with the most prominent features being cardiac rhabdomyoma, CNS and skin involvement, while other characteristics features of TSC (e.g. facial angiofibroma, angiomyolipoma, lymphangioleiomyomatosis) were seen less frequently. In the TOSCA study, TSC was diagnosed at a median age of one year (range 0–69) [11]. The shift towards a younger age at diagnosis seen in our cohort can be attributed to a substantial number of TSC patients with prenatally detected cardiac rhabdomyoma and to the study design that only assessed patients up to 18 years in our survey. The tendency towards a lower age at first diagnosis is consistent with a recently published study by *Davis* et al. in which cardiac rhabdomyoma were the most common initial presenting feature of TSC [23]. Compared to the TOSCA study, in which cardiac rhabdomyoma were found in 34.3% patients, our cohort showed a higher prevalence of cardiac rhabdomayoma in 50% of the patients. Of note and in contrast with findings from the TOSCA registry, arrhythmias/dysrhythmias were also more frequent in our study (5.6% vs. 25.6%). Cardiac rhabdomyoma are highly suggestive of TSC disease [24], mandating further diagnostic work-up in order to establish an early diagnosis. If TSC is diagnosed antenatally, careful monitoring should be implemented using the current international surveillance and management guidelines [10]. Of note, the incidence of cardiac arrhythmias of 25.6% in those with rhabdomyoma (about 13% of all diagnosed patients) in our cohort is high. Moreover, it can be speculated that implementation of a standardized antenatal screening program with fetal ultrasonography and increased awareness for TSC disease has led to an earlier diagnosis – most likely by earlier detection of children with subtle clinical symptoms [11]. Routine antenatal ultrasound examination performed at a gestational age of 19–22 weeks in Germany may possibly miss a certain percentage of ‘late onset’ cardiac rhabdomyoma. In a study by *Bader* et al. the mean gestational age at diagnosis of cardiac rhabdomyoma was 28.4 ± 6.0 weeks (median 28; range 19–37) [24]. However, no serial antenatal ultrasound examinations were performed in this study. Hitherto, there are few off-label studies that reveal effective usage of mTOR inhibition for cardiac rhabdomyoma [25]. Further studies on the natural history of (antenatally detected) cardiac rhabdomyoma in TSC and on possible preventative treatment interventions in severe cases (e.g. critical arrhythmias) are needed, in particular in the light of the unknown prognostic significance of rhabdomyoma associated arrhythmias in TSC.

CNS involvement was the most common clinical symptom at diagnosis in our study. These findings are congruent with the results from *Davis* et al. demonstrating a prevalence of tubers or cortical dysplasia of 94% in their cohort [23] as well as with the results from the TOSCA study (cortical tubers in 82.2%) [11]. The overall prevalence of seizure disorders in our cohort was 69.8%.

With new treatment options (e.g. mTOR inhibitors) for a variety of TSC manifestations (subependymal giant cell astrocytoma (SEGA) [26] and for adjunctive treatment of refractory partial-onset seizures, with or without generalization [27], and renal angiomyolipoma [28]) an early diagnosis and treatment will have a positive impact on the clinical course of children and adolescents with TSC. Moreover, the early use of effective treatment modalities including mTOR inhibitors has not only the potential to ameliorate the clinical course, but also to modify the clinical phenotype (e.g. use of everolimus in EXIST-I and EXIST-II study resulted also in fewer skin involvement). With ongoing changes in diagnostic and therapeutic possibilities, it is imperative to systematically define the spectrum of disease onset. Cutaneous lesions other than hypomelanotic macules (angiofibroma, shagreen patches) is a recognized manifestation, particularly in adult cases [29], and was therefore less frequent in our cohort.

## Conclusions

This is to our knowledge the first population-based estimation of definite or possible TSC incidence in children using current diagnostic criteria, thus providing the medical community with a robust estimate of the incidence of TSC in children. In contrast to our dataset, other studies have reported prevalence rates [12–15] and/or used different diagnostic criteria such as the Roach criteria from 1998 [30] or earlier criteria [13]. Our findings reveal a substantially lower age at first diagnosis of TSC. With the advent and implementation of prenatal imaging the diagnosis of TSC is often made early. In summary, our study demonstrates the presence of cardiac rhabdomyoma in a significant proportion of newly prenatally diagnosed TSC patients. The presence of cardiac rhabdomyoma was associated with cardiac arrhythmias in a substantial number of children in our cohort. Early age at diagnosis will open new avenues to new therapeutic interventions; most importantly early and close EEG monitoring and if abnormal, initiation of early anti-epileptic drug treatment [31, 32], but may also result in earlier use of mTOR inhibitors, thus further modifying the clinical trajectory and phenotype in affected children.

## Additional file

Additional file 1: TSC Questionnaire ESPED Germany. (PDF 75 kb)

## Abbreviations

CNS: Central nervous system
ESPED: German Paediatric surveillance unit for rare diseases
mTOR: mechanistic target of rapamycin
NMI: No mutation identified
TOSCA: TuberOus SClerosis registry to increase disease awareness
TSC: Tuberous sclerosis
