# Correction to: Incidence of tuberous sclerosis and age at first diagnosis: new data and emerging trends from a national, prospective surveillance study

**DOI:** 10.1186/s13023-019-1090-9

**Published:** 2019-05-13

**Authors:** Daniel Ebrahimi-Fakhari, Lilian Lisa Mann, Martin Poryo, Norbert Graf, Rüdiger von Kries, Beate Heinrich, Darius Ebrahimi-Fakhari, Marina Flotats-Bastardas, Ludwig Gortner, Michael Zemlin, Sascha Meyer

**Affiliations:** 1 Department of Pediatric Neurology, Saarland University Medical Center, Building 9, Kirrberger Strasse, 66421 Homburg, Saarland Germany; 2 Department of Pediatric Cardiology, Saarland University Medical Center, Homburg, Germany; 3 Department of Pediatric Oncology and Hematology, Saarland University Medical Center, Homburg, Germany; 4 Division of Epidemiology, Institute of Social Pediatrics and Adolescent Medicine, Ludwig Maximilian’s University, Munich, Germany; 5 German Paediatric Surveillance Unit (ESPED), Coordination Center for Clinical Studies, Heinrich Heine University, Düsseldorf, Germany; 6 Department of Neurology, Boston Children’s Hospital, Harvard Medical School, Boston, MA USA

**Correction to: Orphanet Journal of Rare Diseases (2018) 13:117**

**https://doi.org/10.1186/s13023-018-0870-y**

When calculating the annual incidence rates in article [1], unfortunately an error occurred. The corrected incidence rates should be the following, and the relevant paragraph of the ‘Results’ should therefore correctly read:

Incidence

Based on our findings, the annual incidence rate of TSC (definite or possible TSC) is estimated at a minimum of 1:27.312 live births. However correcting for underreporting using data from previous ESPED analyses, the estimated incidence rate of definite or possible TSC is approximately 1:11.180–1:22.360 live births in Germany.
