# Supplementary material for: Incidence of tuberous sclerosis and age at first diagnosis: new data and emerging trends from a national, prospective surveillance study
Source: Orphanet J Rare Dis. 2018 Jul 17;13:117. doi: 10.1186/s13023-018-0870-y (PMC6050673; doi:10.1186/s13023-018-0870-y)
Supplement: Supplementary file 1 — TSC Questionnaire ESPED Germany. (PDF 75 kb) [file 13023_2018_870_MOESM1_ESM.pdf]

Erhebungseinheit für seltene pädiatrische Erkrankungen in Deutschland

Forschungsstelle für pädiatrische Epidemiologie bei der Deutschen Gesellschaft für Kinder- und Jugendmedizin e.V.

Tuberöse Sklerose Komplex-Erkrankung (TSC) bei Kindern und Jugendlichen

Ziele:

Ermittlung der Inzidenz und Erfassung der klinischen Präsentation gesicherter Fälle der TSC-Erkrankung und der zugrunde liegenden Molekulargenetik bei Kindern <18 Jahren in Deutschland; Erfassung inzidenter Verdachtsdiagnosen („possible diagnosis“ gemäß den revidierten Leitlinien aus dem Jahr 2013; [1]), pränataler (z. B. kardiale Rhabdomyome) und perinatale Besonderheiten und klinischer Auffälligkeiten (z. B. Hautläsionen, wie „White spots“), angewandten Behandlungsstrategien sowie der Einsatz von mTOR-Inhibitoren; Entwicklung einer Konzeption von Interventionsstudien (Fallzahlschätzung, Studienprotokolle).
Darüber hinaus wird die Initiierung eines von ESPED unabhängigen TSC- Registers angestrebt.

Studienleitung:

Prof. Dr. med. Sascha Meyer, Klinik für Allgemeine Pädiatrie und Neonatologie, Universitätsklinikum des Saarlandes, Sektion Neuropädiatrie, 66421 Homburg
Tel.: 06841-1628313, Fax: 06841-1628452, sascha.meyer@uks.eu

Studiendauer: 2 Jahre, Beginn: 01.01.2015

Hintergrund:

Bei der tuberösen Sklerose (TSC) handelt sich um eine genetisch bedingte Multisystemerkrankung (betroffene Gene TSC1 und TSC2), die im Wesentlichen charakterisiert ist durch das Auftreten von Hamartomen im Gehirn, Herz, in Haut, den Augen, Nieren, Lunge und in der Leber. Aufgrund großer Unterschiede der Ausprägung sowie des Auftretens und des Verteilungsmusters der Hamartome zeigt der klinische Phänotyp eine hohe Variabilität. Nicht selten wird die Erkrankung erst im Kleinkindalter – meist nach 3 Jahren – manifest, sodass die klinischen Stigmata nicht immer geeignet sind, sehr früh eine Diagnose zu stellen. Im Jahr 2013 erfolgte eine Aktualisierung und Überarbeitung der Kriterien für die Diagnosestellung sowie der klinischen Überwachung der Patienten mit TSC-Erkrankung [1, 2]. Man geht heutzutage von einer Frequenz von rund 1:6.000 Geburten aus [3, 4].
Bezüglich neurologischer Auffälligkeiten ist zu erwähnen, dass rund 85% der Kinder und Jugendlichen mit TSC unter ZNS-Affektionen leiden (Epilepsie, geistige Entwicklungsretardierung, Verhaltensauffälligkeiten/-störungen, einschließlich Autismus). Epileptische Anfälle können fokaler Natur sein; allerdings kann es auch zum Auftreten eines West-Syndroms mit BNS-Anfällen und problematischer Prognose kommen. Durch das Entstehen und die Größenzunahme subependymaler Riesenzellastrozytome („subependymal giant cell astrocytoma“, SEGA) kann es durch Blockade des Foramen Monroi konsekutiv zu einer Liquorzirkulationsstörung mit hydrozephalem Aufstau und Anstieg des intrakraniellen Drucks kommen.
Die klinische Diagnose einer TSC-Erkrankung kann heutzutage bei rund 85% der Patienten molekulargenetisch gesichert werden (Genort für TSC1 auf Chromosom 9; Genlocus q34 und TSC2 auf Chromosom 16; Genlocus p13.3).

Fragestellungen:

1. Wie hoch ist die Häufigkeit der TSC-Erkrankung in Deutschland?
2. Wie ist die Altersverteilung bei Erstdiagnose? Wie hoch ist der Prozentsatz der Patienten, die bereits pränatal erkannt werden?
3. Ergebnisse der Molekulardiagnostik: Bei wie vielen der Patienten wurde diese durchgeführt und welche Verteilung (TSC1/TSC2) liegt vor?
4. Detaillierte Erfassung der klinischen, organspezifischen Symptomatik und Dysfunktion
5. Gibt es Risikofaktoren für einen schlechten, komplizierten Verlauf (insbesondere Auftreten einer schweren Epilepsie bzw. einer globalen Entwicklungsverzögerung)?
6. Welche Therapieformen werden bei Patienten mit TSC-Erkrankung eingesetzt? Welche antiepileptischen Medikamente (AEDs); wie viele der Patienten erhalten eine Therapieform mit mTOR-Antagonisten? Wie viele operative Therapien (wie z. B. neurochirurgische Resektion eines SEGAs)?

Falldefinition:

Eingeschlossen werden alle Kinder <18 Jahre, die die revidierten Diagnosekriterien für das Vorliegen einer TSC-Erkrankung aus dem Jahr 2013 erfüllen [1].

Gesicherter Fall: *Nachweis einer pathogenetischen TSC1- oder TSC2-Mutation bzw. durch Vorliegen von 2 Hauptkriterien oder 1 Hauptkriterium und ≥ 2 Nebenkriterien;*

Verdachtsfall: *Vorliegen von entweder 1 Hauptkriterium oder ≥ 2 Nebenkriterien*

Hauptkriterien

- Hypomelanotische Flecken (≥ 3 mit einem Mindestdurchmesser von 5 mm)
- Angiofibrome (≥ 3) oder friböser Plaque auf Stirn oder Kopfhaut
- Unguale Fibrome (≥ 2)
- Chagrin-Patch (lederartig verfestigte lumbosakrale Läsion)
- Multiple Netzhauthamartome
- Kortikale Dysplasien
- Subependymale Knötchen
- Subepend. Riesenzellastrozytome (SEGA)
- Kardiale Rhabdomyome
- Lymphangioleiomyomatose
- Angiomyolipome (≥ 2)

Nebenkriterien

- Konfettiartige Hautläsionen
- Zahnschmelzdefekte (≥ 3)
- Gingivale/enorale Fibrome
- Retinaler achromatischer Fleck
- Multiple Nierenzyten
- Nichtrenale Hamartome

Logistik:

Bitte melden Sie jeden Erkrankungsfall nach obiger Falldefinition an ESPED. Danach erhalten Sie einen Fragebogen zur Erfassung der wichtigsten klinischen, genetischen und epidemiologischen Angaben (Rücksendung an ESPED-Zentrale). Darüber hinaus erhalten Sie einen Brief mit der Bitte um Weitergabe an die Eltern. Darin werden die Eltern um Einwilligung zur Teilnahme an einer Verlaufserfassung gebeten, die mittels standardsiertem Telefoninterview durchgeführt wird.

Literatur:

1. Krueger DA, Northrup H; International Tuberous Sclerosis Complex Consensus Group. Pediatr Neurol 2013; 49:255-65
2. Northrup H, Krueger DA; International Tuberous Sclerosis Complex Consensus Group. Pediatr Neurol 2013; 49:243-54
3. Yilmaz U, Altmeyer K, Meyer S. Radiologe. 2013; 53:1091-8
4. O'Callaghan F, Shiell A, Osborne J, Martyn C. Lancet 1998; 352:318–319
